# The Predictive Accuracy of the New Trauma Score and the Revised Trauma Score in Predicting the Mortality of Patients Presenting to the Emergency Department of a Tertiary Care Hospital in Karachi

**DOI:** 10.7759/cureus.76421

**Published:** 2024-12-26

**Authors:** Subas Ali, Talha Bhatti, Shehzadi Rimsha, Rabia Masroor Hashmi, Sumayah Khan, Waqas Rind, Raja Muhammad Mussab

**Affiliations:** 1 General Surgery, Civil Hospital Karachi, Karachi, PAK; 2 Neurosurgery, Civil Hospital Karachi, Karachi, PAK; 3 Surgery, University Hospitals Birmingham NHS Trust, Birmingham, GBR; 4 Orthopaedics and Trauma, Jinnah Postgraduate Medical Centre, Karachi, PAK; 5 Orthopaedics and Trauma, Russell Hall Hospital, Dudley, GBR

**Keywords:** emergency medicine and trauma, general trauma surgery, new trauma score, revised trauma score, trauma centre

## Abstract

Background

Trauma is a major public health issue, causing disease and death globally. Injuries can range from mild to severe, requiring different levels of medical attention from a skilled team.

Objectives

To predict the accuracy of the new trauma score (NTS) and the revised trauma score (RTS) for predicting the mortality of patients presenting in the emergency department of a tertiary care hospital in Karachi.

Methods

A descriptive cross-sectional study was conducted in the Emergency Department of Civil Hospital Karachi (CHK), Pakistan, for six months and included 366 patients. Patients with blunt, traumatic, crush, or penetration injuries were included in this study. All patients were given a score according to the NTS and RTS.

Results

The median age of the study's participants was 31.90 ± 7.46 years. A mortality rate of 4.64% was noted. The NTS demonstrated a diagnostic accuracy of 97.5%, a sensitivity of 88.2%, a specificity of 98%, a positive predictive value (PPV) of 68.2%, and a negative predictive value (NPV) of 99.4% for predicting mortality. The RTS exhibited a diagnostic accuracy of 99.1%, a sensitivity of 88.2%, a specificity of 99.7%, a PPV of 93.8%, and an NPV of 99.4%. When the NTS and RTS were combined, they exhibited a diagnostic accuracy of 97.5%, a sensitivity of 82.5%, a specificity of 98%, a PPV of 68.2%, and an NPV of 99.4% for predicting mortality.

Conclusion

As a result, we conclude that both RTS and NTS were equally useful in predicting hospitalization, morbidity, the need for mechanical ventilation, and mortality.

## Introduction

Trauma-related injuries are the most well-known external injuries that pose a threat to worldwide public health. Statistics show that injuries account for 16% of the global illness burden and that traumatic injuries account for over 4.5 million fatalities each year [1,2]. Additionally, 90% of injury-related fatalities occur in lower-middle-income nations [2]. According to World Health Organization (WHO) data, workplace injuries, personal accidents, falls from heights, and traffic accidents are the main causes of trauma [3]. By 2030, traffic-related fatalities will be the fifth leading cause of mortality worldwide [4,5]. In underdeveloped countries, trauma ranks first among the four leading causes of death and accounts for the majority of life that is lost [6]. Trauma severity, physiological reserve, prompt revival, and adequate care all have an impact on the outcomes of severe injuries [7].

Pre-hospital treatment depends on the initial evaluation and the severity of injury since it determines the ultimate hospital and the level of trauma care that will be offered [8]. When pressed for time during intense situations, it can be difficult to appropriately assess the extent of the damage [9]. Pre-hospital trauma assessment is a vital step in delivering the right patient to the right hospital in the quickest possible time, hence early intervention counts as a crucial part of the complete healthcare approach in these circumstances [10]. A scoring system is frequently used in pre-hospital trauma triage systems to guide patients to the best course of treatment [11]. Incorrect prioritization bears consequences of both under- and over-triaging. A low-sensitivity assessment tool indeed exhibits a significant percentage of false-negative scenarios, which may indicate that a severe injury's diagnosis and treatment were unsuccessful. Improper triage has been associated with a greater fatality rate [9]. On the other hand, insufficient specificity is associated with a high proportion of false positive instances, or over-triage [8]. Less injured patients receive treatment at high-level medical facilities, which puts an unnecessary strain on hospital resources and increases the likelihood of adverse events for simultaneously treated non-trauma patients [12,13].

Recent years have seen the development of various trauma scores. The trauma scoring system can be useful both in emergency rooms and at the scene of the injury. This grading method aids in determining the result and, consequently, the patient's management. One of them is the revised trauma score (RTS), which is currently very popular [14,15]. The Glasgow Coma Scale (GCS) coded value, respiratory rate (RR), and systolic blood pressure (SBP) are variables that are monitored. It has a sensitivity of 99% and specificity of 62% with 7 as the cut-off value [14]. Even though RTS is extensively used, it is regarded as an outdated scoring system for predicting trauma morbidity and mortality, leading to the development of a new trauma score (NTS) and a revision of RTS [16]. According to the literature, NTS is thought to be more accurate than RTS at predicting morbidity and mortality. The actual score of GCS rather than the code of GCS, a change to SBP value used as a coded value, and finally the usage of saturation of oxygen (SpO2) rather than RR are all included in the NTS. It is regarded as a key technique in the early management of trauma. The sensitivity of NTS is 95%, and its specificity is 82%; the cutoff value is 18 [16].

This study aimed to predict the accuracy of both scores for predicting the mortality of trauma patients presenting in the emergency department of Civil Hospital Karachi (CHK), as international studies have been conducted for the initial assessment of patients presenting in the emergency department using this scoring system. No such data were available in Pakistan and demographically, the population is different. The score should be easy to use by any healthcare professional, cost-effective, require no laboratory tests, or complex studies, be time-saving, and aid in making an early decision for the benefit of the patient with good accuracy.

## Materials and methods

A descriptive cross-sectional study was commenced in the Emergency Department of CHK, Pakistan, after approval from the Institutional Review Board of the institute. The duration of the study was six months from 22 August 2020 to 22 February 2021. The sample size was calculated by using the sensitivity and specificity Calculator, taking statistics of NTS sensitivity as 95% and specificity as 82% [16]. The estimated prevalence of trauma in our tertiary care hospital was taken as 20% [17]. A sample size of 366 was achieved with a 95% confidence interval and desired precision of 5%. A consecutive sampling technique was used.

Inclusion and exclusion criteria

All trauma patients between 13 to 60 years of age presenting to the emergency department, of either gender, and with all types of injuries like blunt, road traffic accidents, and crush or penetration injuries were included in this study. Patients discharged from the emergency or transferred to another emergency department, patients on antihypertensive drugs, anticoagulation, and antiplatelets, known chronic liver disease (CLD) with coagulopathy, patients with an altered level of consciousness due to causes other than trauma (like drugs or alcohol), and patients who received cardiopulmonary resuscitation (CPR) during the prehospital phase were excluded from the study. The effect modifiers were based on the patient’s history as given by accompanying attendants and the medical records available. Effect modifiers included patients who suffered from a stroke in the last six months, with a known case of pulmonary disease (COPD), patients taking steroids or any other anti-asthmatic drug, patients not previously diagnosed as hypertensive who were diagnosed on presentation, and patients with a history of cardiac disease and taking antianginal drugs.

All patients meeting the inclusion criteria were enrolled in the study by the principal investigator after obtaining informed and written consent. Patients’ parameters (demographic details, GCS, RR, SpO2, SBP) and effect modifiers via proper history and examination were assessed by the doctor receiving the patients in the emergency department. All patients were given a score according to the NTS and RTS. The data were recorded in the Proforma, and every patient was assessed and managed accordingly. They were followed after 24 hours, and the outcome was noted as alive or dead. The outcome was compared with two scores till discharge from the hospital to provide the accuracy of the scores.

The RTS is an important scoring system that uses patients presenting GCS coded value, SBP, and RR. It is calculated by the formulas: RTS=0.9368 GCS code value+0.7326 SBP coded value+0.2908 RR coded value. The cut-off value of the revised trauma score is <7 and will be considered a predictive value for mortality.

New trauma score (NTS)

It is a modification of RTS. The parameters are GCS, SBP coded value, and SPO2 coded value. It is calculated by the formula: NTS= GCS +(0.2983 * SBP _NTS_ coded value) + (0.8709 * SpO2_ NTS _coded value). The cut-off value of NTS is <18, which was considered a predictive value for mortality.

For data analysis, IBM SPSS Statistics for Windows, Version 23 (Released 2015; IBM Corp., Armonk, NY, United States) was utilized. For the quantitative variables (GCS, SpO2, RR, SBP, NTS, and RTS), mean and standard deviation were employed. Age and gender were used as qualitative variables, and their frequency and percentages were calculated. The sensitivity, specificity, PPV and NPV, and diagnostic accuracy were calculated using a 2x2 table. Through stratification, potential effect modifiers, such as age, gender, and comorbidity (hypertension and diabetes mellitus), were found. After stratification, a 2x2 table was utilized for PPV, NPV, specificity, sensitivity, and diagnostic accuracy. The chi-square test was also employed and a p-value below 0.05 was regarded as significant.

## Results

A total of 366 patients suffered blunt, traumatic, crush, or penetration injuries. The patients had a median age of 31.90 ± 7.46 years, and their characteristics can be found in Table 1. Of the patients, 274 (74.86%) were male while 92 (25.14%) were female as mentioned in Table 2. There were 5.74% hypertensive cases, and 5.19% had diabetes mellitus.

**Table 1:** Descriptive statistics of characteristics of patients GCS: Glasgow Coma Scale, SBP: systolic blood pressure, DBP: diastolic blood pressure, RR: respiratory rate, SaO2: saturation of oxygen, RTS: revised trauma score, NTS: new trauma score

Variables	Mean	95% Confidence Interval for Mean	
		Minimum	Maximum
Age (Years)	31.90±7.46	31.13	32.67
GCS	14.21±2.45	13.96	14.46
SBP (mmHg)	107.98±14.34	106.50	109.45
DBP (mmHg)	71.61±11	70.58	72.65
RR (normal: 12-18/minute)	21.68±4.27	21.24	22.12
SaO2 (normal: 95-100%)	97.90±1.07	97.79	98.01
RTS score	7.69±1.02	7.59	7.80
NTS score	18.74±2.82	18.45	19.03

**Table 2:** Gender and co-morbidities

	Frequency, n (N:366)	Percentage
Gender		
Male	274	74.86%
Female	92	25.14%
Comorbidities		
Hypertension	21	5.74%
Diabetes mellitus	19	5.19%

Our findings indicated that NTS had 88.2% sensitivity, 98% specificity, 68.2% PPV, 99.4% NPV, and 97.5% diagnostic accuracy for predicting mortality. Similarly, RTS had 88.2% sensitivity, 99.7% specificity, 93.8% PPV, 99.4% NPV, and 99.1% diagnostic accuracy, as presented in Table 3.

**Table 3:** Accuracy of the new trauma score and the revised trauma score for predicting mortality PPV: positive predictive value; NPV: negative predictive value

New trauma score and revised trauma score		Mortality		Total	Sensitivity	Specificity	PPV	NPV	Accuracy
		Yes	No						
New trauma score	<18	15	7	22	88.2%	98%	68.2%	99.4%	97.5%
	≥18	2	342	344					
	Total	17	349	366					
Revised trauma score	<7	15	1	16	88.2%	99.7%	93.8	99.4%	99.1%
	≥7	2	348	350					
	Total	17	349	366					

Combining NTS and RTS resulted in 88.2% sensitivity, 98.9% specificity, 78.9% PPV, 99.4% NPV, and 98.4% diagnostic accuracy for predicting mortality, as demonstrated in Table 4.

**Table 4:** Accuracy of combined new trauma score and the revised trauma in predicting mortality PPV: positive predictive value; NPV: negative predictive value

New Trauma Score	Revised Trauma Score	Mortality		Sensitivity	Specificity	PPV	NPV	Accuracy
		Yes	No					
<18	<7	30	8	88.2%	98.9%	78.9%	99.4%	98.4%
≥18	≥7	4	690					

A statistically significant association was seen between combined new and revised trauma scores to predict mortality in ages less than and greater than 35 years, both genders, hypertensive, non-hypertensive, and non-diabetes mellitus patients (p < 0.05). Details are described in Table 5.

**Table 5:** Association of combined new and revised trauma score to predict mortality in different ages, genders, and with different comorbidities *chi-square test of association; ** significant when ≤ 0.05

	New Trauma Score	Revised Trauma Score	Mortality		Total	ꭓ2*	p-value**
			Yes	No			
≤35 years of age	<18	<7	6	3	9	131.91	0.0005
	≥18	≥7	2	260	262		
>35 years of age	<18	<7	9	4	13	62.71	0.0005
	≥18	≥7	0	82	82		
Male	<18	<7	11	7	18	135.49	0.0005
	≥18	≥7	2	254	256		
Female	<18	<7	4	0	4	171.52	0.0005
	≥18	≥7	0	88	88		
Hypertensive	<18	<7	7	1	8	17.06	0.0005
	≥18	≥7	0	13	13		
Non-hypertensive	<18	<7	8	6	14	152.55	0.0005
	≥18	≥7	2	329	331		
Diabetic mellitus	<18	<7	5	6	11	0.833	0.633
	≥18	≥7	2	6	8		
Non-diabetic mellitus	<18	<7	10	1	11	314.52	0.0005
	≥18	≥7	0	336	336		

Our stratification analysis showed that the diagnostic accuracy of the scores was high in all stratified groups, as shown in Table 6. These results confirm the reliability and effectiveness of NTS and RTS in predicting mortality for patients suffering from blunt, trauma, crush, or penetration injuries.

**Table 6:** Accuracy of combined new trauma score and revised trauma score in predicting mortality based on age, gender, and co-morbidities PPV: positive predictive value; NPV: negative predictive value

		Sensitivity	Specificity	PPV	NPV	Accuracy
Age	For ≤35 years	75%	98.9%	66.7%	99.2%	98.2%
	For >35 years	100%	95.3%	69.2%	100%	95.7%
Gender	For male	84.6%	88.2%	61.1%	99.2%	96.72%
	For female	100%	10%	100%	100%	100%
Comorbidities	For hypertension	100%	92.9%	87.5%	100%	95.2%
	For non-hypertension	80%	98.2%	57.1%	99.4%	97.7%
	For diabetic mellitus	71.4%	50%	45.5%	75%	57.8%
	For non-diabetic mellitus	100%	99.7%	90.9%	100%	99.7%

## Discussion

This research aimed to determine the effectiveness of NTS and RTS in predicting the mortality of patients who presented to the emergency department with blunt, traumatic, crush, or penetration injuries. A total of 366 trauma patients aged 13-60 years were included. The average age of patients was 31.90 ± 7.46 years. When analyzing gender differences in injured patients to enhance the accuracy of trauma-mortality predicting scores, males were found to have more chances of trauma as compared to females. This was consistent with other studies where males faced frequent traumatic injuries [3,16,18-21]. In contrast, a study conducted in France evaluated a total of 1,112 trauma patients and found that the majority of them were females [22]. As urbanization, motorization, industrialization, and change in socioeconomic values had become part of our lives, females also had to have equal participation with males as the breadwinners of the house in our society, and this activity involved traveling on roads making both genders equally vulnerable to injury.

Our research found that RTS had a sensitivity, specificity, PPV, NPV, and accuracy of 88.2%, 99.7%, 93.8%, 99.4%, and 99.1%, respectively, whereas NTS had a sensitivity, specificity, PPV, NPV, and diagnostic accuracy for predicting mortality of 88.2%, 98%, 68.2%, 99.4%, and 97.5%, respectively. Manoochehry et al. found that the sensitivity estimate for RTS was 82% and the specificity estimate was 91%. The PPV estimate was 8.9% while the NPV estimate was 20%. The accuracy estimate was 95% [2]. In another study, Yii-Ting Huang and colleagues used RTS as a predictor of mortality. They found that the PPV was 9.9% and the NPV was 99.9%, with an odds ratio (OR) of 94.72 [23]. Cassignol et al. utilized triage-RTS as a diagnostic tool to predict in-hospital mortality. The results showed a sensitivity of 91%, specificity of 35%, PPV of 10%, and NPV of 98% [22].

In a prospective observational study, Jeong and colleagues utilized data from a trauma registry of a tertiary hospital to compare the mortality predictive abilities of NTS and other trauma scores. The study found that NTS had a higher accuracy in predicting in-hospital mortality than RTS, with a specificity of 95% and a sensitivity of 82% [16], which is in contrast to what we found in our study. In determining hospital mortality, Amini et al. discovered that NTS had a sensitivity rate of 99% and a specificity rate of 94%. Additionally, they found that RTS had 98% sensitivity and 97% specificity in the same study [20]. The same study compared NTS with RTS and found OR values greater than 10. Intending to discover something new, we combined both scores only to find out that diagnostic accuracy rose to 98.4%, which is better than the accuracy of NTS itself, in the current study. Hence, we applied this combination against the factors of age, both genders, and hypertensive, non-hypertensive, and non-diabetes mellitus patients to find a statistically significant (<0.05) association in predicting mortality, which, to the best of our knowledge, has not been found out in the present literature.

The association between age and mortality in trauma patients has been studied previously. A study done to see in-hospital mortality with increasing age showed a rising slope of mortality after the age of 35 years. This could be due to altered physiologic reserves and the presence of co-morbidities, which become apparent with increasing age [24]. As in Pakistan, the average age of lifespan is 60 years, we have not included the population above it because usually, the geriatric age group is a separate extreme-of-age group with multiple comorbidities and anticoagulant use can adversely affect prognosis. On the other hand, age groups below 35 years are more exposed to road traffic accidents making them more prone to trauma-related mortality. Hence, the use of trauma-mortality predicting scores comes to be utilized again to good effect. Hypertension is a well-known and significant risk factor for increased mortality, especially in trauma patients and traumatic brain injuries. We have not classified patients as per their mechanism of injury but needless to say, in the triage phase, it is difficult to rule out traumatic brain injuries [16]. We have also seen a significant association of normotensive patients with combined RTS and NTS scores, which is unique and in contrast to the literature and raises further questions on the mechanism or nature of their trauma presentation.

According to the authors, when it comes to pulse oximetry, using NTS was a much more practical way to go than trying to determine RR per minute [20]. Predictive performance was compared to triage-RTS with NTS in a study conducted in France. The area under the curve (AUC) difference between both was 0.06 with a p-value of less than 0.01. The study found NTS was significantly better at predicting mortality in trauma patients [22]. A systematic review found that the NTS had a sensitivity of 82% and a specificity of 86% when mortality was used as a reference, indicating good performance. SBP, GCS, and SpO2 are readily accessible physiological measures on which NTS is based [8]. Numerous studies have demonstrated that, even when an electronic instrument is utilized, the measurement of RR in the field or emergency room is erroneous because of patient circumstances, psychological considerations, and loud noises [25]. Additionally, when a patient is intubated immediately on the scene, RR is frequently not recorded. SpO2 is a straightforward metric that can be used to monitor peripheral oxygenation because it is objective and unambiguous. According to a meta-analysis finding, using the Northern French Alps Trauma System (TRENAU) in conjunction with NTS appeared to be a good alternative for pre-hospital triage in a system that included doctors' and nurses’ presence. High-quality pre-hospital care was given in the best possible way in this combination with on-site expert evaluation [8].

Limitations

Our research had some drawbacks. Because this study only involved one center, generalizations might not be accurate. Therefore, external validation should be carried out in various demographics and nations. Only adults qualified as the target demographic. Therefore, patients under the age of 13 might find that this score is not helpful. Separate research is required for the creation and implementation of a trauma scoring system for the pediatric population due to the distinctive physiological characteristics of pediatric patients.

## Conclusions

Trauma scores are useful tools for determining the prognosis of patients and assisting with initial triage. We concluded that both the RTS and NTS scores had similar predictive accuracy; however, due to their parameters, NTS could be easily applied in our setup for predicting mortality. Both NTS and RTS were accurate in predicting morbidity and mortality in hypertensive patients. Since NTS was straightforward and probably feasible, we advise maximization of its research in pre-hospital and emergency triage, treatment evaluation, and injury research. By doing this, trauma care might be significantly improved in locations with known resource shortages or evidence-free triage procedures, which is typical in many parts of low- and middle-income nations like Pakistan.

## References

[1] Wärnberg Gerdin L, Khajanchi M, Kumar V (2020). Comparison of emergency department trauma triage performance of clinicians and clinical prediction models: a cohort study in India. BMJ Open.

[2] Manoochehry S, Vafabin M, Bitaraf S, Amiri A (2019). A comparison between the ability of revised trauma score and Kampala trauma score in predicting mortality; a meta-analysis. Arch Acad Emerg Med.

[3] Khari S, Zandi M, Yousefifard M (2022). Glasgow Coma Scale versus physiologic scoring systems in predicting the outcome of ICU admitted trauma patients; a diagnostic accuracy study. Arch Acad Emerg Med.

[4] Türkdoğan FT, Coşkun A (2021). Evaluation of epidemiological factors of radiological imaging methods in thoracoabdominal trauma patients. Eurasian J Emerg Med.

[5] Mishra A, Tripathi A, Chaudhary S, Gupta R, Pradhan P (2021). Study of trauma patients in the emergency department of a tertiary care hospital in north India during covid 19 pandemic. International Journal of Health and Clinical Research.

[6] Nik A, Sheikh Andalibi MS, Ehsaei MR, Zarifian A, Ghayoor Karimiani E, Bahadoorkhan G (2018). The efficacy of Glasgow Coma Scale (GCS) score and Acute Physiology and Chronic Health Evaluation (APACHE) II for predicting hospital mortality of ICU patients with acute traumatic brain injury. Bull Emerg Trauma.

[7] Llompart-Pou JA, Chico-Fernández M, Sánchez-Casado M (2017). Scoring severity in trauma: comparison of prehospital scoring systems in trauma ICU patients. Eur J Trauma Emerg Surg.

[8] Gianola S, Castellini G, Biffi A (2021). Accuracy of pre-hospital triage tools for major trauma: a systematic review with meta-analysis and net clinical benefit. World J Emerg Surg.

[9] van Rein EAJ, Houwert RM, Gunning AC, Lichtveld RA, Leenen LPH, van Heijl M (2017). Accuracy of prehospital triage protocols in selecting severely injured patients. A systematic review. J Trauma Acute Care Surg.

[10] Bigham BL, Bull E, Morrison M, Burgess R, Maher J, Brooks SC, Morrison LJ (2011). Patient safety in emergency medical services: executive summary and recommendations from the Niagara Summit. CJEM.

[11] van Rein EA, van der Sluijs R, Houwert RM, Gunning AC, Lichtveld RA, Leenen LP, van Heijl M (2018). Effectiveness of prehospital trauma triage systems in selecting severely injured patients: Is comparative analysis possible?. Am J Emerg Med.

[12] Dehli T, Uleberg O, Wisborg T (2018). Trauma team activation - common rules, common gain. Acta Anaesthesiol Scand.

[13] Jones CM, Cushman JT, Lerner EB (2016). Prehospital trauma triage decision-making: a model of what happens between the 9-1-1 call and the hospital. Prehosp Emerg Care.

[14] Champion HR, Sacco WJ, Carnazzo AJ, Copes W, Fouty WJ (1981). Trauma score. Crit Care Med.

[15] Kondo Y, Abe T, Kohshi K, Tokuda Y, Cook EF, Kukita I (2011). Revised Trauma scoring system to predict in-hospital mortality in the emergency department: Glasgow Coma Scale, age, and systolic blood pressure score. Crit Care.

[16] Jeong JH, Park YJ, Kim DH (2017). The new trauma score (NTS): a modification of the revised trauma score for better trauma mortality prediction. BMC Surg.

[17] Hyder AA, Razzak JA (2013). The challenges of injuries and trauma in Pakistan: an opportunity for concerted action. Public Health.

[18] Paudel S, Dhungana S, Pokhrel N, Dhakal GR (2021). Epidemiology of trauma patients presented at emergency department of trauma center. J Nepal Health Res Counc.

[19] Mohammed Z, Saleh Y, AbdelSalam EM, Mohammed NB, El-Bana E, Hirshon JM (2022). Evaluation of the revised trauma score, MGAP, and GAP scoring systems in predicting mortality of adult trauma patients in a low-resource setting. BMC Emerg Med.

[20] Amini K, Abolghasemi Fakhri S, Salehi H, Bakhtavar HE, Rahmani F (2021). Mortality prediction in multiple trauma patients using GAP, RTS and NTS models. Trauma Mon.

[21] Gang MC, Hong KJ, Shin SD (2019). New prehospital scoring system for traumatic brain injury to predict mortality and severe disability using motor Glasgow Coma Scale, hypotension, and hypoxia: a nationwide observational study. Clin Exp Emerg Med.

[22] Cassignol A, Markarian T, Cotte J (2019). Evaluation and comparison of different prehospital triage scores of trauma patients on in-hospital mortality. Prehosp Emerg Care.

[23] Huang YT, Huang YH, Hsieh CH, Li CJ, Chiu IM (2019). Comparison of injury severity score, Glasgow Coma Scale, and revised trauma score in predicting the mortality and prolonged ICU stay of traumatic young children: a cross-sectional retrospective study. Emerg Med Int.

[24] Lee JH, Kim MJ, Hong JY, Myung J, Roh YH, Chung SP (2021). The elderly age criterion for increased in-hospital mortality in trauma patients: a retrospective cohort study. Scand J Trauma Resusc Emerg Med.

[25] Philip KE, Pack E, Cambiano V, Rollmann H, Weil S, O'Beirne J (2015). The accuracy of respiratory rate assessment by doctors in a London teaching hospital: a cross-sectional study. J Clin Monit Comput.

